# CCL7 Is a Negative Regulator of Cutaneous Inflammation Following *Leishmania major* Infection

**DOI:** 10.3389/fimmu.2018.03063

**Published:** 2019-01-08

**Authors:** Jill Ford, Angela Hughson, Kihong Lim, Susana V. Bardina, Wuyuan Lu, Israel F. Charo, Jean K. Lim, Deborah J. Fowell

**Affiliations:** ^1^Department of Microbiology and Immunology, David H. Smith Center for Vaccine Biology and Immunology, Aab Institute of Biomedical Sciences, University of Rochester, Rochester, NY, United States; ^2^Department of Microbiology, Icahn School of Medicine at Mount Sinai, New York, NY, United States; ^3^Department of Biochemistry and Molecular Biology, Institute of Human Virology, University of Maryland School of Medicine, Baltimore, MD, United States; ^4^Department of Medicine, Cardiovascular Research Institute, University of California, San Francisco, San Francisco, CA, United States

**Keywords:** chemokine, CCL7, skin, inflammation, neutrophils, macrophage, parasite, *Leishmania major*

## Abstract

The chemokine CCL7 (MCP3) is known to promote the recruitment of many innate immune cell types including monocytes and neutrophils to sites of bacterial and viral infection and eosinophils and basophils to sites of allergic inflammation. CCL7 upregulation has been associated with many inflammatory settings including infection, cardiovascular disease, and the tumor microenvironment. CCL7's pleotropic effects are due in part to its ability to bind numerous chemokine receptors, namely CCR1, CCR2, CCR3, CCR5, and CCR10. CCL7-blockade or CCL7-deficiency is often marked by decreased inflammation and poor pathogen control. In the context of *Leishmania major* infection, CCL7 is specifically upregulated in the skin one-2 weeks after infection but its role in *L. major* control is unclear. To determine CCL7's impact on the response to *L. major* we infected WT and CCL7^−/−^ C57BL/6 mice. *L. major* infection of CCL7-deficient mice led to an unexpected *increase* in inflammation in the infected skin 2 weeks post-infection. A broad increase in immune cell subsets was observed but was dominated by enhanced neutrophilic infiltration. Increased neutrophil recruitment was associated with an enhanced IL-17 gene profile in the infected skin. CCL7 was shown to directly antagonize neutrophil migration *in vitro* and CCL7 add-back *in vivo* specifically reduced neutrophil influx into the infected skin revealing an unexpected role for CCL7 in limiting neutrophil recruitment during *L. major* infection. Enhanced neutrophilic infiltration in CCL7-deficient mice changed the balance of *L. major* infected host cells with an increase in the ratio of infected neutrophils over monocytes/macrophages. To determine the consequence of CCL7 deficiency on *L. major* control we analyzed parasite load cutaneously at the site of infection and viscerally in the draining LN and spleen. The CCL7^−/−^ mice supported robust cutaneous parasite control similar to their WT C57BL/6 counterparts. In contrast, CCL7-deficiency led to greater parasite dissemination and poor parasite control in the spleen. Our studies reveal a novel role for CCL7 in negatively regulating cutaneous inflammation, specifically neutrophils, early during *L. major* infection. We propose that CCL7-mediated dampening of the early immune response in the skin may limit the ability of the parasite to disseminate without compromising cutaneous control.

## Introduction

The rapid recruitment of innate effector populations such as monocytes, macrophages, and neutrophils to the site of infection provides immediate pathogen control in many infectious settings. Innate recruitment is driven by pathogen-induced tissue expression of a variety of chemokines and cytokines that facilitate innate cell extravasation into the tissue and mobilization from the bone marrow. For pathogens that benefit from residency in the host, the recruited innate cells can also serve as cellular reservoirs of infection. Thus, the recruited monocytes and neutrophils can mediate direct pathogen killing but may also facilitate pathogen persistence and/or spread. It is likely that the magnitude and composition of the early innate infiltrate will influence the balance between pathogen control and persistence.

Members of the monocyte chemoattractant protein (MCP) family play central roles in the recruitment of innate effectors to sites of inflammation (1–4). CCL2 (MCP-1) and its main receptor CCR2 have been widely studied and linked to a host of inflammatory responses including infection, cardiovascular disease, neuro-inflammatory and degenerative disease, autoimmunity, and cancer. CCL7 (MCP-3) mediates effects on a host of innate and adaptive immune cell types through binding to numerous receptors including CCR1, CCR2, CCR3, CCR5, and CCR10 (5). In many settings, CCL7 genetic deletion or CCL7 blockade reduces innate immune cell recruitment, limits inflammation and renders mice more susceptible to infectious challenge (6–11). Correspondingly, CCL7 overexpression in tumors enhances recruitment of macrophages, dendritic cells, neutrophils, and natural killer cells and slows tumor growth (12–14). In contrast to their agonist actions, the function of MCPs are regulated by proteolytic cleavage that can results in antagonist activity (15–17). Matrix metalloproteinase cleaved-CCL7 binds to CCR1, 2, and 3 receptors but does not elicit signaling and dampens inflammation *in vivo* (16, 18, 19). In addition, high affinity binding of native CCL7 to CCR5 blocks receptor signaling and antagonizes CCR5 activation by CCL4 (MIP-1β) (20). Thus, disease-specific enhancement of CCL7 expression may lead to either agonist or antagonist activity depending on the local milieu.

Cutaneous infection with the protozoan parasite *Leishmania major* (*L. major*) is marked by the rapid influx of neutrophils within hours of infection (21, 22). This early neutrophil response is transient and its role in preventing or promoting parasite infection remains controversial (23–25). Neutrophils can harbor live parasites which can be transferred to tissue macrophages through uptake of infected apoptotic neutrophils or by direct parasite uptake after release from lytic neutrophils (26). *L. major* resists immune control through interference with a number of macrophage and dendritic cell functions in the infected dermis that can limit innate pathogen control and modulate T cell activation (27). Ultimately, the production of cytokines by CD4^+^ T cells is responsible for local control of infection (28, 29). Production of IFNγ activates macrophages for iNOS-dependent intracellular parasite killing that controls local parasite load and limits dissemination (30). In susceptible mouse strains, the dominance of T cell-derived IL-4 and IL-13, and the paucity of IFNγ- producing Th1 cells, prevents local parasite control and is associated with wide-spread parasite dissemination (31–34).

*L. major* can actively modulate the availability of chemokine signals for immune cell recruitment to the infection site (35). Specifically, parasite downregulation of CXCR3 chemokines (CXCL9,10) and CCL5 limits the recruitment of leishmaniacidal IFNγ-producing Th1 cells (36). In contrast, CCL7 is one of a limited number of chemokines to be upregulated specifically in the *L. major* infected dermis compared to other dermal proinflammatory stimuli (35). The role of CCL7 in *L. major* infection is not known. In humans, elevated expression of CCL7 has been associated with a late cutaneous stage of *L. major* infection that is dominated by lymphocytes and macrophages while granulocytes are poorly represented (37). Given CCL7s ability to bind to a host of different receptors on numerous immune cell types, and the possibility of acting as a chemokine antagonist, its impact on tissue immunity to infection is likely to be highly context and stage dependent.

In this current study, the use of CCL7-deficient mice (38) reveals a striking role for CCL7 in limiting the magnitude of local cutaneous inflammation. The enhanced dermal inflammation early in infection in the absence of CCL7 was characterized by elevated neutrophils, IL-4, and IL-17. CCL7 add-back specifically limited neutrophil recruitment to the infection site highlighting an unexpected role for CCL7 in controlling neutrophil numbers in the *L. major* infected dermis. Moreover, despite local control of *L. major* in the dermis, CCL7 deficient mice had slightly elevated parasite burdens in the dLN early in the infection and a striking increase in parasite load in the spleen late in infection. Thus, CCL7 plays an important role in limiting cutaneous inflammation following *L. major* infection and appears critical for visceral parasite control.

## Materials and Methods

### Mice

WT BALB/c and C57BL/6 mice were purchased from National Cancer Institute. CCL7^−/−^ mice were provided by Dr. Israel Charo (UCSF) and further backcrossed to N12 C57BL/6.

### *Leishmania major* Infection

Mice were infected intradermally in the ear with 2 × 10^5^ infectious (PNA-selected) *Leishmania major* promastigotes (strain WHOM/IR/-/173) in 10 μl PBS as previously described (39). Parasite titres were determined by limiting dilution analysis. Briefly, ear suspensions were resuspended in 450 μl cRPMI. Fifty microliters of the suspension was plated in a 96 well flat bottom plate containing 150 μl of the parasite growth medium, HOSMEM II. 10-fold dilutions were carried out across the wells of the plate and titers were determined at 6 and 10 days post plating. *L. major*-RFP parasites (DsRed MHOM/IL/80/Friedlin) were provided by Dr. David Sacks (NIAID) (26).

### Tissue Processing

Individual ears were split into dorsal and ventral sides and placed in 1 mL of 1 mg/mL collagenase/dispase (Roche) containing 1 mM CaCl_2_ and incubated for 30 min with agitation at 37°C. After 30 min, EDTA was added to a final concentration of 5 mM and ears were returned to 37° for another 5 min to complete dissociation. Ears were gently pushed through a strainer and washed twice with HBSS/2% FCS. Draining LNs (cervical) were gently pushed through a strainer and cells isolated.

### Gene Expression

RNA from whole ear homogenates was isolated using the RNeasy Fibrous tissue kit from Qiagen. cDNA was synthesized using the Life Technologie's High Capacity cDNA Reverse Transcription kit. Samples were loaded onto TLDA cards and RT-PCR performed using 7900HT Fast Real-Time PCR System (Applied Biosystems) at the University of Rochester Genomics Center. HPRT and GAPDH were used as endogenous controls to normalize input cDNA and one WT sample used as the calibrator.

### Protein Quantification

Individual ears were incubated in 500 μl of collagenase/dispase containing 1 mM CaCl2 as above, gently pushed through a strainer using a minimal volume of PBS to rinse strainer. Samples were centrifuged at 10,000 rpm and supernatants were collected for ELISA. Murine MCP-3 ELISA development Kit was purchased from Peprotech and assay was performed according to manufacturer's protocol.

### Flow Cytometry

The following antibodies were used: anti-Ly6G/Ly-6C (RB6-8C5), anti-CD103 (2E7), APC anti-CD11b (M1/70), anti-Ly6C (HK1.4), anti-Ly6G (1A8), anti-F4/80 (BM8), and MHC-II (M5/114.15.2) from Biolegend; anti-CD11c (N418), anti-FcεR1a (MAR-1), anti-CD49b (DX5), and anti-CD4 (RM4-5) from Ebioscience; anti-CD45 (30-F11) from BD Biosciences; Live/Dead Aqua Fixable Dead Cell stain from Life Technologies.

### Cytokine Analysis by ELISPOT

Single cell suspensions from ear tissue and draining lymph nodes were cultured on anti-cytokine mAb-coated elispot plates (Millipore) in the presence or absence of soluble Leishmania antigen (SLA) (equivalent to 1 × 10^6^ parasites/ml) overnight. Plates were developed as previously described (39).

### *In vitro* Neutrophil Migration Assay

Neutrophils were freshly isolated from mouse bone marrow using the EasySep Neutrophil enrichment kit (STEMCELL). Cell migration chambers (Millicell EZ slide eight-well glass, Millipore) were coated with 5 μg/ml recombinant mouse ICAM-1 with or without the indicated chemokines. For *in vitro* live cell imaging, neutrophils were placed in L15 medium (Invitrogen) in the chamber at 37°C, and video microscopy was conducted using TE2000-U microscope (Nikon) with × 20 magnification. Migration analysis was performed using Volocity software (Quorum).

### CCL7 Add Back

Synthetic active CCL7 (pCCL7) and control inactive CCL7 (Ctrl) peptides were prepared as described (6). *L. major* infected WT C57BL/6 or CCL7^−/−^ mice were injected i.v. with 500 ng of synthetic active CCL7 peptide or control peptide 2 times a day on days 9, 10, 11, 12, and 13 post infection for a total of 10 injections (6). Mice were euthanized and tissues harvested on day 14.

### Statistical Analysis

Student *t*-test, Mann Whitney test, one way ANOVA with multiple comparisons or two way ANOVA were performed where indicated: ^*^p ≤ 0.05, ^**^p ≤ 0.01, ^***^p ≤ 0.001, ^****^p ≤ 0.0001.

### Ethics Statement

All animal experimentation in this study was reviewed and approved by the University Committee on Animal Resources (UCAR), the University's Institutional Animal Care and Use Committee (IACUC) under protocol “UCAR-2006-145R.” The University of Rochester and its animal research facilities are fully accredited by the Association for Assessment and Accreditation of Laboratory Animal Care, International, and adheres to the humane use as dictated by the Animal Welfare Act, and “The Guide For the Care and Use of Laboratory Animals.” PHS Assurance number is D16-00188 (A3292-01).

## Results

### CCL7 Induction Following *L. major* Infection

**Figure 1:**
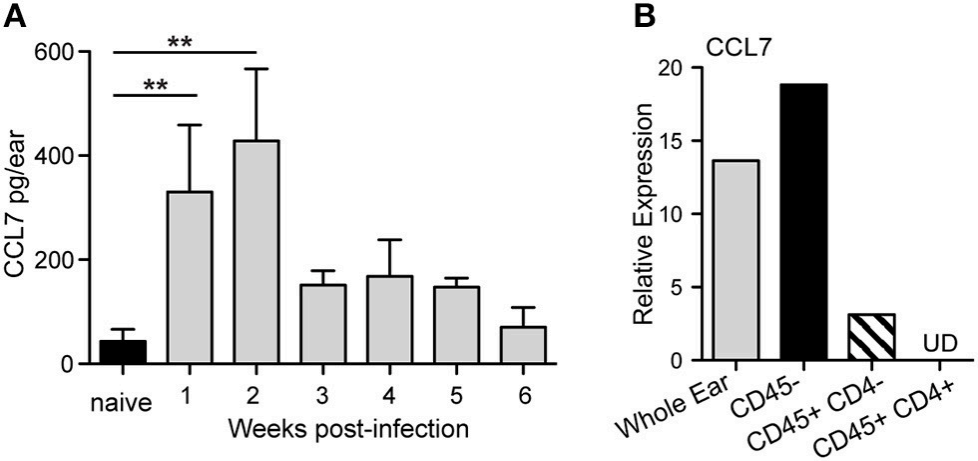
CCL7 expression following *L. major* infection. WT C57BL/6 mice were infected with *L. major* intradermally in the ear. **(A)** Ear tissue was homogenized at indicted times post-infection and the supernatants assayed for CCL7 protein by ELISA. Statistics by Mann Whitney. Four to six mice per group, two independent experiments. **(B)** Cells were isolated from 2 week *L. major*-infected ears, sorted into the respective cell populations and mRNA isolated. CCL7 mRNA measured by RT-PCR, normalized to HPRT, and expression levels calibrated to whole ear cell suspensions from uninfected mice. Results from mRNA pooled from 4 to 6 mice, one representative experiment of 2–3 independent comparable experiments. UD, undetected. ^**^p ≤ 0.01.

Our previous work assessed the regulation of chemokines in the *L. major* infected dermis and found that both CCL1 and CCL7 were specifically upregulated by *L. major* in the infected dermis (35). While CCL1 was upregulated directly in the infected macrophage (35), CCL7 upregulation was detected from mRNA extracts of unfractionated infected skin tissue, but not from mRNA of infected macrophages, suggesting its induction in non-infected immune and/or stromal cells in the infected skin. To dissect the regulation of early host expression of CCL7 we initially determined the kinetics of CCL7 induction. Elevated CCL7 protein expression at the infection site peaked 1 and 2 weeks post-infection (Figure 1A). Basic fractionation of cells in the infected ear, revealed CCL7 was predominantly expressed by CD45- non-hematopoietic cells in the skin (Figure 1B) consistent with observations of cutaneous fibroblasts being a potent source of CCL7 (40, 41). Thus, CCL7 is upregulated within the tissue stroma 1–2 weeks post-*Leishmania* infection.

### Enhanced Inflammation in the *L. major*-Infected Skin in the Absence of CCL7

**Figure 2:**
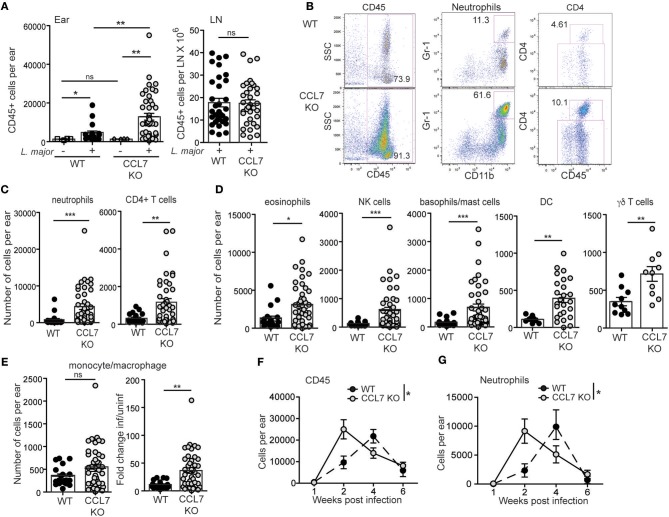
Enhanced inflammation at the *L. major* infection site in the absence of CCL7. WT and CCL7 KO mice were infected with *L. major* in the ear and immune cells in the infected tissue 2 weeks post-infection characterized by flow cytometry. **(A)** Leukocyte counts (CD45^+^ cells) in the uninfected (– *L. major*) and infected (+ *L. major*) ears (left panel) and draining LN (right panel) of C57BL/6 WT and CCL7 KO mice. **(B)** Representative FACS plots from 2 week-infected ears. Numbers in the FACS plots represent % of total cells for CD45 and % of CD45^+^ cells for neutrophils and CD4. **(C)** Neutrophils and CD4 T cell numbers in the ear 2 weeks post-infection. **(D)** Phenotypic analysis of CD45^+^ cells types, numbers of cells per ear 2 weeks post-infection. **(E)** Left, monocyte numbers per ear of 2 week infected mice; right, fold change relative to uninfected. Symbols represent individual mice, 4–6 mice per group, pooled from at least 4 independent experiments. Statistics by Mann Whitney. For all cell types in **(C,D)** *P* < 0.05 WT vs. CCL7 KO for additional paired analysis of the means of individual experiments (*n* = 4) by Wilcoxon matched-pairs rank test. **(F,G)** Kinetic analysis of inflammation in the ear following *L. major* infection. **(F)** Total number of CD45^+^ cells per ear by flow cytometry and **(G)** number of neutrophils per ear by flow cytometry. 4–5 mice per group per timepoint, 3 independent experiments. Statistics by two way ANOVA. ^*^p ≤ 0.05, ^**^p ≤ 0.01, ^***^p ≤ 0.001.

To determine the role of CCL7 in the immune response to *L. major* we infected WT and CCL7 deficient (CCL7 KO) C57BL/6 mice (38) with *L. major* and first analyzed the recruitment of immune cell subsets to the infected skin. Similar to previously published work on CCL7 deficient mice (6, 38), uninfected (steady state) CCL7 KO mice had a specific reduction in the frequency and number of monocytes in circulation, and in the spleen and skin (Supplementary Figure 1), but representation of other major immune cell subsets was similar to WT (6, 38), including neutrophils (Supplementary Figure 1). Steady state CD45 numbers in the uninfected skin were comparable between WT and CCL7 KO (Figure 2A). Two weeks post-infection, when CCL7 expression peaked at the infection site in WT mice (Figure 1A), the CCL7 KO mice exhibited a striking 4- to 5-fold increase in immune infiltration into the infected skin (Figures 2A,B). In contrast, WT and CCL7 KO cell numbers in the draining lymph node (LN) following *L. major* infection were comparable (Figure 2A, right panel). Phenotypic analysis of the CD45^+^ cells in the ear indicated that most immune cell types were elevated in the infected ear of CCL7 KO mice (Figures 2B–E). Neutrophils were the dominant immune cell type, with CD4^+^ T cells and eosinophils also major contributors to the infiltrate (Figures 2B–D). Monocyte/macrophage numbers in the infected ear were not significantly different between WT and CCL7 KO (Figure 2E, left panel). However, consistent with the reduced starting number of monocytes in the CCL7 KO (Supplementary Figure 1), there remained a significant and robust increased fold change in monocyte numbers in the infected ears compared to uninfected ears in the absence of CCL7 (mean fold change in monocyte numbers in the infected skin/uninfected skin: 11.2 for WT BL/6, 36.52 for CCL7 KO BL/6), similar to the other immune cell types (Figure 2E, right panel). Therefore, monocytes/macrophages can be recruited to the *L. major* infected skin independently of CCL7. The phenotypic distinction between neutrophils (CD11b^+^ Gr-1^hi^) and monocytes/macrophage (CD11b^+^ Gr-1^lo/+^) was confirmed with additional analysis of expression of Ly6C and Ly6G: neutrophils were CD11b^+^ Ly6C^+^ Ly6G^hi^ while monocytes/macrophage were CD11b^+^ Ly6C^+^ Ly6G^lo^, (Supplementary Figure 2). Moreover, the CD11b^+^ Gr-1^hi^ neutrophils were negative for F4/80 and MHC Class II expression (Supplementary Figure 2). The heightened inflammatory response was not associated with a paucity of regulatory T cells: similar numbers, and frequency within the CD4 compartment, of Foxp3^+^ regulatory T cells were found at the infection site of CCL7 KO and WT mice (mean/SEM number/ear: WT 45.46 ± 11.7, CCL7 KO 48.54 ± 17.03). The enhanced accumulation of immune cells at the site of infection in the CCL7 KO was also not associated with a compensatory increase in expression of other monocyte chemoattractant proteins (MCPs) CCL2, CCL8, CCL12 compared to infected WT mice (Supplementary Figure 3). Therefore, CCL7 deficiency results in an unexpected enhancement in inflammation at the *L. major* infection site, suggesting CCL7 regulates the overall magnitude of the local immune response. These results are counter to the reported early decrease in immune infiltration into the infected brain following West Nile Virus (6) and decreases in inflammation on CCL7-blockade. Indeed, the unexpected augmented inflammatory response appears to be specific to *L. major* infection as no enhancement was seen in the size of the immune response in the ear on challenge with protein antigen and adjuvant (CFA or Alum) or on infection with *C. albicans* (Supplementary Figure 4). Kinetic analysis of the local immune response in the *L. major* infected ear revealed the immune changes to be remarkably transient. The increase in number of CD45^+^ cells and all respective subset analysis had resolved by week 4 of infection (Figures 2F,G), similar to the kinetics of CCL7 expression in WT mice (Figure 1).

**Figure 3:**
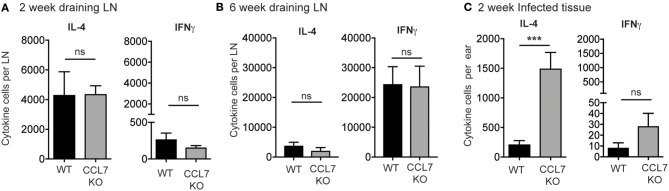
Elevated number of *L. major*-specific IL-4-producing cells in the infected ear in the absence of CCL7. WT and CCL7 KO mice were infected with *L. major* in the ear and cytokine-producing cells in the infected ear and draining LN analyzed by *L. major*-specific ELISPOT. Cytokine producing cells per LN 2 weeks post-infection **(A)** and 6 weeks post-infection **(B)**. **(C)** Cytokine producing cells per infected ear 2 weeks post-infection. ^***^p ≤ 0.001.

We next determined the cytokine response associated with the elevated inflammation. The 2-week *L. major* infection timepoint is marked by an early CD4 T cell response that is dominated by IL-4 producing cells, with very few IFNγ producing cells, in both resistant (C57BL/6) and susceptible (BALB/c) mouse strains (35, 42–44). To determine if the loss of CCL7 alters the function of the developing CD4 T cell response to *L. major*, we performed *L. major* specific ELISPOTs from the dLN. CCL7-deficiency did not alter the generation, magnitude or cytokine balance of *L. major-*specific effector T cells in the dLN, with an early IL-4 dominance observed in both WT and CCL7 KO mice (Figure 3A) and a later Th1 protective response with predominant IFNγ production in both B6 and CCL7 KO mice (Figure 3B). However, at the 2 week timepoint of enhanced inflammation, the number of IL-4 producing cells, but not IFNγ producing cells, was significantly enhanced in the infected tissue in the CCL7 KO mice (Figure 3C). Thus, CCL7 deficiency appears to enhance the efficiency of early IL-4-producing effector T cell recruitment and/or retention at the infection site. A similar enhancement in IL-4 was seen in a lung model of fungal infection when CCL7 was neutralized (45). However, IL-4 does not appear to be the driver of the heightened inflammation as blockade of IL-4 (anti-IL-4 mAb, 11B11) did not alter the enhanced leukocytic infiltration (data not shown). At later timepoints after infection, the anti-leishmania T cell response in CCL7 KO C57BL/6 mice resembled that of their WT C57BL/6 counterparts with a predominance of IFNγ producers (Figure 3B) suggesting CCL7 is not required for the priming of a protective anti-leishmania Th1 response.

### Enhanced Neutrophilic Inflammation Is Associated With Increased Expression of IL-17 at the Infection Site

**Figure 4:**
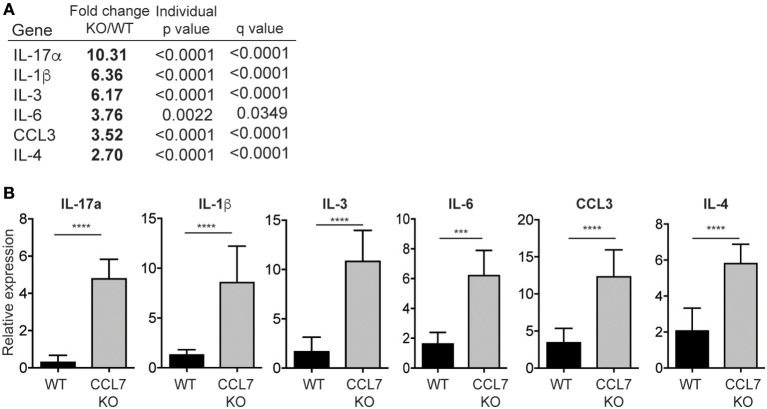
Neutrophil-promoting cytokines and chemokines upregulated following *L. major* infection in the absence of CCL7. mRNA was isolated from whole ear extracts of WT and CCL7 KO mice 10 days post-infection and expression of 94 inflammatory genes tested using a custom gene array micro-fluidics card (see Supplementary Figure 2 for gene list). **(A)** Fold change for all genes that were significantly differentially expressed between WT and CCL7 KO mice. Statistics by two way ANOVA, corrected for multiple comparisons with a 5% FDR (Benjamini, Frieger, and Yekutieli). **(B)** Relative gene expression for all genes significantly up-regulated in the absence of CCL7. Three to four mice per group, representative data from one of two independent experiments. ^***^p ≤ 0.001, ^****^p ≤ 0.0001.

To identify possible mechanisms that facilitate the enhanced inflammation in the absence of CCL7 we analyzed cellular and gene expression changes in whole-ear extracts 10 days after *L. major* infection, immediately preceding the enhanced inflammatory infiltrate. Flow cytometry, 10 days post-infection, revealed that neutrophils were the only immune population to be significantly elevated in CCL7 KO mice at this time point (mean/SEM number/ear: WT 122.6 ± 44.11, CCL7 KO 501.6 ± 124.5, *p* ≤ 0.05) suggesting infiltration of neutrophils precedes the more general increase in immune recruitment at 2 weeks. We screened for changes in expression of 94 inflammatory genes (including chemokines, cytokines, and trafficking molecules) between WT and CCL7 KO infected mice using a custom gene array micro-fluidics card (36) (gene list, Supplementary Figure 5). Surprisingly, of the 94 genes tested, just 6 genes were significantly up-regulated and CCL7 was the only significantly downregulated gene in the CCL7 KO environment compared to WT (Figures 4A,B). The up-regulated genes IL-17a, IL-3, IL-6, IL-1β, and CCL3 are all associated with Type 17 inflammation and important drivers of local recruitment and expansion of neutrophils (46). Indeed, genetic ablation of IL-17 has been shown to reduce neutrophilic infiltration in *L. major* infected tissues (25, 47). IL-4 was also upregulated confirming the elevated IL-4 producing cells on Day 14 as measured by ELISPOT (Figure 3C). Thus, the absence of CCL7 induction early after *L. major* infection triggers a local IL-17 milieu in the skin that is consistent with the enhanced recruitment of neutrophils.

### CCL7 Negatively Regulates Neutrophil Migration and Recruitment to the *L. major* Infection Site

CCL7 is often associated with the positive regulation of myeloid cell recruitment to sites of inflammation and infection. In contrast, we found that in the absence of CCL7 there was enhanced recruitment of many immune cell types suggesting CCL7 may directly or indirectly dampen the magnitude of immune cell recruitment in some inflammatory/infectious settings.

**Figure 5:**
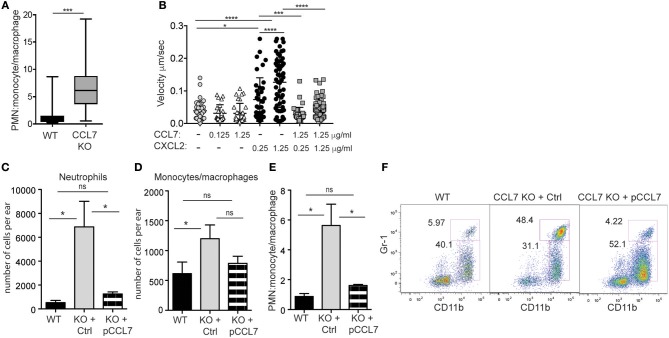
CCL7 negatively regulates neutrophilic accumulation in the *L. major*-infected dermis. Analysis of neutrophil (PMN) to monocyte/macrophage ratios in the 2 week *L. major*-infected skin by flow cytometry. **(A)** PMN:monocyte/macrophage ratio based on cell number. Four to six mice per group mice from at least four independent experiments. Statistics by Mann Whitney. **(B)** Live imaging of neutrophil migration on ICAM-1 coated-plates in response to indicated chemokines, at designated μg/ml. Three independent experiments. Statistics by one way ANOVA. **(C–F)** CCL7 add-back. Control, inactive (Ctrl), or active (pCCL7) CCL7 peptides were administered i.v. twice daily for 5 consecutive days starting day 9 post-infection and ear tissue harvested 2 weeks post-infection. Three to four mice per group, representative data from one of two independent experiments. **(C)** Number of neutrophils in infected ear. **(D)** Number of monocytes/macrophages in infected ear. **(E)** PMN:monocyte/macrophage ratio. **(C–E)** Statistics by one way ANOVA. **(F)** Representative flow cytometry. Numbers represent % of all CD45^+^ cells in the respective gates. ^*^p ≤ 0.05, ^***^p ≤ 0.001, ^****^p ≤ 0.0001.

In particular, the unexpected increase in neutrophil accumulation within the 2-week *L. major* infected dermis (Figure 2) resulted in the ratio of neutrophils to monocytes/macrophages being heavily skewed in favor of neutrophils (Figure 5A). This altered balance in neutrophil and monocyte/macrophage accumulation in the infected skin of CCL7 KO mice could be a consequence of the loss of CCL7 as a positive regulator of monocyte/macrophage recruitment or due to the loss of a previously undescribed CCL7-dependent pathway that antagonizes neutrophil recruitment. We focused on the possible role of CCL7 in negatively regulating neutrophil migration/recruitment *in vitro* and *in vivo*.

*In vitro*, we compared neutrophil migration to CCL7 and CXCL2, a canonical chemotactic factor for neutrophils. By live-imaging (48), we examined the migration of freshly isolated neutrophils on ICAM-1 coated surfaces in the presence or absence of immobilized chemokines, alone or in combination. CXCL2 drove neutrophil migration in a dose-dependent manner, while CCL7 was poorly chemotactic (Figure 5B). However, when combined, the presence of CCL7 significantly inhibited neutrophil migration to CXCL2 (Figure 5B). These data suggest that CCL7 may directly alter the migration of neutrophils to sites of inflammation.

*In vivo*, to directly test the role of CCL7 in shaping the immune infiltrate in the infected tissue, we used synthetic active peptides of CCL7 to add-back CCL7 function to CCL7 KO mice (6). Control or active CCL7 peptides were administered twice daily as described (6) from day 9 to day 13 post infection, around the time of CCL7 induction in WT mice. CCL7 add-back led to an acute reduction in the number of neutrophils at the infection site (Figures 5C,F), with minimal changes in the number of monocytes/macrophages (Figures 5D,F). This change in the number of neutrophils, restored the neutrophil:monocyte/macrophage ratio in the infected ear to that seen in WT mice (Figures 5E,F). Thus, unexpectedly, CCL7 creates a tissue environment that appears to limit neutrophil accumulation in the context of cutaneous *L. major*. Although the magnitude of the differences between WT BL/6 and CCL7 KO was less in the spleen, neutrophils were also elevated in the spleen on *L. major* infection, relative to monocytes/macrophages, and the add-back of CCL7 significantly reduced neutrophil accumulation (Supplementary Figure 6), reinforcing a negative regulatory role for CCL7 in neutrophil accumulation in tissues.

### Loss of CCL7 Results in Local Cutaneous Parasite Control but Enhanced Visceral Parasite Load

**Figure 6:**
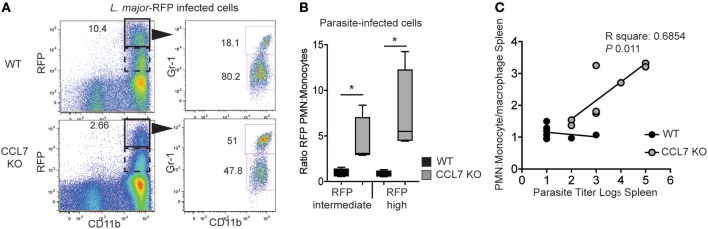
CCL7 deficiency skews the cellular host for *L. major* in favor of the neutrophil. *L*. major-RFP were used to phenotype the host cells harboring *L. major* in the infected ear. WT and CCL7 KO mice were infected intradermally with *L. major*-RFP and the phenotype of RFP^+^ host cells determined by flow cytometry 2 weeks post-infection. **(A)** Representative flow cyometry plots. Left panels, RFP by CD11b expression and gate used to further phenotype RFP^hi^ cells (solid line) and RFP^int^ (dashed line); right panels, Gr-1 and CD11b expression of the RFP^hi^ cells with numbers indicating the frequency of RFP^hi^ cells with these markers. **(B)** Ratio of the number of RFP^int^ and RFP^hi^ PMN to monocytes/macrophages. Three to four mice per group, data from three independent experiments. Statistics by one way ANOVA. **(C)** Linear regression of PMN:monocyte/macrophage ratio and parasite titer in the spleen. (R square: 0.077 WT and 0.685 CCL7 KO. *P*-value: 0.507 WT and 0.011 CCL7 KO). ^*^p ≤ 0.05.

We next wanted to determine the physiological consequence of CCL7-deficiency on parasite control. The cellular distribution of *L. major* is known to change over time: neutrophils being the dominant host cell type hours after infection and monocyte/macrophages emerging as the predominant host cell type 6–7 days post-infection (26). This switch in cellular host has evoked mechanisms where infected-neutrophils “hand over” parasites to macrophages and/or DC by infected-neutrophil engulfment or direct parasite uptake following neutrophil lytic release (23, 26, 49). To determine the impact of the CCL7-deficiency on *L. major* host cell distribution, we used fluorescently-tagged *L. major*-RFP (26). Two weeks post-infection, the RFP^hi^ cells in the infected WT ear were primarily CD11b^hi^ Gr-1^lo^ monocytes/macrophages (Figure 6A), as previously described (26). In contrast, CD11b^hi^ Gr-1^hi^ neutrophils remained the predominant RFP^+^ cells in the infected ear of CCL7-deficient mice (Figure 6A). We observed a heterogeneous pattern on RFP expression by infected cells, with a population of RFP^hi^ cells (gated in Figure 6A) and a population with a lower RFP signal, RFP^int^ cells. Analysis of both the RFP^hi^ and RFP^int^ populations showed, in the absence of CCL7, the balance of infected host cell types was skewed in favor of the neutrophil (Figure 6B). To determine if this change in *L. major* host cell type may influence the ability of the CCL7 KO mouse to control parasite growth, we directly compared the neutrophil:monocyte ratio with the splenic parasite load in individual CCL7 KO and WT BL/6 mice. We found a striking positive correlation between enhanced neutrophil:monocyte ratios and the inability to control parasites in the spleen (Figure 6C).

**Figure 7:**
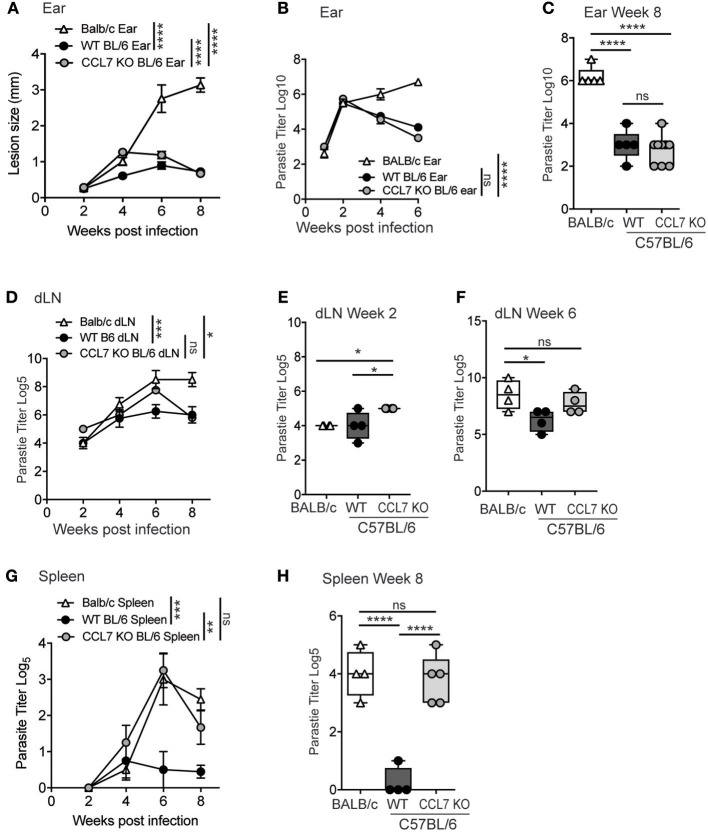
CCL7 deficiency maintains local parasite control but results in enhanced parasite load in the spleen. **(A)** WT BALB/c, WT C57BL/6 (BL/6), and CCL7 KO C57BL/6 (BL/6) mice were infected with *L. major* in the ear pinna and the lesion size measured weekly. Statistics by two way ANOVA. **(B)** Kinetic analysis of parasite load in the infected ear by limiting dilution analysis. Statistics by two way ANOVA. **(C)** Parasite load in the ear 8 weeks post infection. Statistics by one way ANOVA. **(D)** Kinetic analysis of parasite numbers in the draining LN by limiting dilution analysis, statistics by two way ANOVA. Parasite load in the dLN at 2 weeks **(E)** and 6 weeks **(F)** by limiting dilution analysis, statistics by one way ANOVA. **(G)** Kinetic analysis of parasite load in the spleen following cutaneous infection in the ear pinna, by limiting dilution analysis. Statistics by two way ANOVA. **(H)** Parasite load in the spleen 8 weeks post-infection in the footpad. Representative data from 1 of 3 independent experiments, 4–6 mice per group per timepoint per experiment. Statistics by one way ANOVA. ^*^p ≤ 0.05, ^**^p ≤ 0.01, ^***^p ≤ 0.001, ^****^p ≤ 0.0001.

C57BL/6 mice are genetically resistant to *L. major* infection, locally controlling parasite numbers through a robust IFNγ-dependent immune response, and limiting parasite visceralization as measured in the spleen. In contrast, BALB/c mice are genetically susceptible to *L. major* infection and fail to control parasite numbers cutaneously and viscerally in the spleen, due to the induction of a dominant IL-4 response to the pathogen (28, 29). Given the robust infiltration of immune cells into the cutaneous site of infection (Figure 2), the appearance of parasites in the spleens of CCL7 deficient mice on the C57BL/6 background was unexpected (Figure 6C). To examine parasite control in more detail, we infected WT and CCL7 deficient (CCL7 KO BL/6) C57BL/6 mice with *L. major* subcutaneously in the ear and assessed the cutaneous lesion size over time and the parasite load locally in the skin and viscerally in the dLN and spleen. We included susceptible WT BALB/c mice as a positive control for elevated parasite burdens in the skin and spleen. CCL7 KO mice had an increased lesion size compared to WT C57BL/6 control mice 4 weeks after infection that was resolved by 8 weeks (Figure 7A), suggesting a possible delay in the ability to control parasite growth in the skin. However, kinetic analysis of cutaneous parasite loads using limiting dilution analysis showed control of parasite load in the skin of CCL7 KO that was comparable to their WT counterparts (Figures 7B,C). Thus, the increased lesion size at 4 weeks in the absence of CCL7 was not due to enhanced parasite load and may be a consequence of the altered cutaneous inflammatory response. To determine early dissemination of the parasite we analyzed parasite burdens in the skin-draining LN (dLN) over time post infection (Figures 7D–F). Temporal changes in dLN parasite load between CCL7 KO and WT C57BL/6 mice were not significantly different and both KO and WT C57BL/6 mice had controled parasite numbers by 8 weeks compared with BALB/c mice (Figure 7D). On more detailed analysis we found modest but significantly increased parasite numbers (~5-fold) in the dLN (2 week Figure 7E; 6 week Figure 7F) of mice lacking CCL7, suggesting enhanced dissemination from the skin. Parasites were first detected in the spleen at 4 weeks post-infection in all groups (Figure 7G). While WT C57BL/6 mice prevented further parasite growth in the spleen, CCL7 KO mice had significantly more parasites in the spleen at 6 and 8 weeks post-infection compared to WT C57BL/6, with levels approximating those seen for susceptible WT BALB/c mice (Figure 7G). To confirm this striking observation, we infected mice in an alternate subcutaneous site, the footpad. Similar to ear infection, the size of the footpad lesions and parasite loads in the footpad were not significantly different in the CCL7 KO mice compared to WT C57BL/6 mice throughout the 8 week timecourse (data not shown), but the CCL7 KO C57BL/6 mice remained unable to control parasite loads in the spleen (Figure 7H). Therefore, the absence of CCL7 does not alter the ability of mice to control parasite growth cutaneously (Figure 7C), but CCL7 appears critical for parasite control viscerally, as highlighted in the spleen (Figure 7H).

## Discussion

We reveal a novel role for CCL7 in limiting cutaneous inflammation in response to infection with the protozoan parasite *Leishmania major*. In the absence of CCL7, mice exhibited enhanced dermal inflammation 2 weeks post-infection that was associated with an elevated IL-17 gene signature and an increase in neutrophil recruitment. CCL7 add-back reduced the local accumulation of neutrophils at the infection site suggesting a specific role for CCL7 in limiting neutrophil influx into infected tissues. Indeed, *in vitro*, we found that CCL7 could directly antagonize neutrophil migration. Disrupting CCL7 had important consequences for parasite control: cutaneous parasite control remained intact but there was a failure to contain parasites in the spleen. Thus, host induction of CCL7 in response to *L. major* infection appears critical for limiting neutrophilic inflammation in the first few weeks of infection and also protects against visceral parasite growth.

Our results are counter to a number of studies where CCL7 blockade mitigates inflammation and suggest that in certain settings *in vivo* CCL7 induction can be associated with antagonist activity that dampens inflammation. Most inflammatory and infectious studies have linked CCL7 to the promotion of inflammation with positive effects on recruitment of many innate immune cell types including neutrophils, NK cells, monocytes and DC (6, 10, 13, 50–53), and basophils and eosinophils (11, 54, 55). The balance of CCL7 agonist and antagonist activity with respect to leukocyte recruitment will likely depend on a variety of conspiring events in the local inflammatory milieu (56–59). The degree of pathogen or tissue leukocyte-driven proteolytic cleavage will regulate the degree of degradation vs. liberation of cleaved CCL7 with antagonist activity (16, 18). Indeed, the Leishmania metalloprotease GP63 is an extensively studied parasite virulence factor (60) and could directly alter chemokine activity. Competition between chemokines in the milieu for binding chemokine receptors and/or glycosaminoglycans (GAGs) may also regulate net chemotactic activity in the infected tissue. Differences between CCL7's monomeric binding to GAGs compared to oligomerizing CCL2 may change relative availability/duration and/or agonist activity (61). A number of natural chemokine antagonists have been described (56), including CCL7 that can bind to CCR5 and block CCL4 activity (20). Here we show that CCL7 can also antagonize neutrophil migration in response to CXCL2, despite no evidence for CCL7 binding to the CXCL2 receptor, CXCR2. How CCL7 mediates this effect will require additional mechanistic studies.

It is unclear how CCL7 negatively regulates cutaneous inflammation in the context of *L. major*. Our preliminary characterization of the cell types involved suggests the main source of CCL7 in the *L. major*-infected skin is non-hematopoietic stromal cells (Figure 1) (35) that are not directly *L. major*-infected suggesting CCL7 induction is indirect, triggered by other host factors in response to infection, possibly through IL-1β or IFNs (62, 63). CCL7 has also been associated with Type 2 responses (64) being linked to basophil and eosinophil recruitment and/or activation in allergic inflammation and has recently been shown to be induced by IL-33 (65). CCL7 induction early in *L. major* infection when IL-4 dominates the infected cutaneous milieu in both genetically susceptible and resistant mouse strains (35, 42–44) is consistent with such Type 2 instruction. However, what is unusual about our studies is the apparent role CCL7 plays in *reducing* the magnitude of inflammation in the *L. major* infected skin. This could be mediated through a direct negative effect on neutrophils themselves through active antagonism of chemokine receptors on infiltrating neutrophils (as seen *in vitro*). CCL7 may also work indirectly, by promoting the accumulation of myeloid cells to the infection site (66, 67) that might compete for, or inhibit, signals that enhance neutrophil accumulation. For example, in response to mucosal infection with *Toxoplasma gondii* incoming inflammatory monocytes directly inhibited neutrophil accumulation and activation at the site of infection and blocking monocyte recruitment led to severe neutrophil-dependent pathology (68). Additional indirect exacerbation of neutrophil accumulation in the absence of CCL7 could come from impaired neutrophil clearance due to reduced number or function of recruited monocytes/macrophage (69).

CCL7-deficiency reveals an unusual disconnect between robust parasite control in the skin and a failure to control parasite load in the spleen. At present, we are unclear if these two events are linked. The increased parasite load in the spleen in absence of CCL7 could arise due to increased dissemination from the skin and/or could be due to an enhanced ability of the parasites to proliferate in the spleen once there. Our analysis of parasite load in the draining LN suggests that initial dissemination to the LN may be enhanced in the absence of CCL7 and this effect might be subsequently amplified in the spleen. We failed to detect differences in *L. major* numbers in the spleen of CCL7 KO mice early after cutaneous infection, despite using an additional sensitive qPCR-based approach, suggesting that early seeding of the spleen does not appear to explain the elevated numbers late in infection. CCL7 may drive the recruitment of distinct monocyte subsets and/or numbers to the two tissues and these monocytes may differ in their ability to support or attenuate parasite growth as host cells or as drivers of protective Th1 immunity (70). In response to infection with a highly viscerally-prone Leishmania strain such as *L. donovani*, infection induces emergency hematopoiesis with the expansion of non-classical myeloid progenitors. These myeloid cells have a regulatory phenotype, are more permissive to infection and appear to facilitate parasite persistence in the spleen (71, 72). Indeed, we cannot rule out the possibility of our neutrophil or monocyte/macrophage populations containing myeloid-derived suppressor cells, indistinguishable by phenotypic analysis (73), which could account for the impaired parasite control in the spleen. Alternatively, CCL7 may recruit the same monocyte populations to both sites but differ in the negative regulation of neutrophil recruitment to specific tissues, where neutrophils can act as a “safe haven” for parasites (74, 75). Thus, there may be CCL7-dependent location-specific differences in immune requirements for clearance or in permissiveness of parasite growth (25). Future characterization of the splenic innate compartment following *L. major* infection in the presence and absence of CCL7 should shed light on local immune changes that are unique to the spleen.

In addition to possible CCL7 requirements specifically in the spleen, it is worth speculating on whether the enhanced cutaneous inflammation seen in the absence of CCL7 may play a direct role in enhancing the transport of *L. major* out of the skin to visceral sites. Despite intensive study of the host response to *L. major* in the infected dermis, the mechanisms whereby *L. major* exits the dermis and disseminates to visceral organs are poorly understood. Parasites could disseminate via lymphatic drainage or be carried out by motile host cells. The transient increase in the magnitude of cutaneous immune infiltration seen in the absence of CCL7, dominated by neutrophils, raises the possibility that this milieu provides increased cellular targets that aid in leishmania dissemination. Indeed, susceptibility to infection is reduced when neutrophil influx is limited via IL-17 deficiency (47). As suggested from *in vivo* imaging studies (26) and early immunohistological studies (21), neutrophils contain live parasites and appear to contribute little to the direct elimination of *L. major*. Rather, neutrophils may act as tissue reservoirs for *L. major* with lytic release of parasites promoting local lymphatic drainage and/or hand-off to other immune cells in the skin such as macrophages and DCs (25, 26, 49, 74, 76). Interestingly, recent measures of neutrophil life-span, particularly in inflammatory settings, have challenged the notion that neutrophils are short-lived (77), raising the possibility that neutrophils could represent a more long-term harbor for *L. major* and/or act as “slow release” vehicles at the infection site extending the timeframe for possible dissemination. In addition, neutrophil reverse migration back out of tissues, either via lymphatics (78–81) or retrograde transendothelial entry back into the bloodstream (82–84), provides a potential mechanism whereby neutrophils can directly transport *L. major* between tissues. Despite this attractive idea, we have been unable to detect parasite-containing cells in the peripheral blood of *L. major* infected WT or CCL7 KO mice. Nevertheless, host induction of CCL7 may limit the magnitude of the cutaneous immune response to protect against visceral disease by reducing the number of cellular “pathogen-transporters.”

Chemokines are inheritantly promiscuous and often play redundant roles in leukocyte recruitment to damaged or infected tissues. Characterization of disease-specific chemokine profiles have helped to define context-specific chemokine milieus, yet the functional contribution of single (or combinations of) chemokine players is unclear (56). Recently, CXCR2 deficient mice were shown to have a counterintuitive exaggerated inflammatory response linked to imbalanced myeloid cell populations (85). Our studies also highlight that a chemokine such as CCL7 that is usually associated with enhancing inflammation has an unexpected regulatory role in limiting cutaneous inflammation in the context of *L. major* infection, and specifically in negatively regulating neutrophil tissue accumulation. Limiting cutaneous inflammation following *L. major* infection via CCL7 may have potential therapeutic application for locally controlling the inflammation at the infection site and attenuating visceral parasite growth.

## Author Contributions

JF, AH, KL, and DF designed the experiments. JF, AH, and KL performed the experiments. SB, WL, IC, JL, and KL provided reagents and advice on experimental design and data interpretation. JF and DF wrote the manuscript. AH, SB, JL, and DF edited the manuscript.

### Conflict of Interest Statement

The authors declare that the research was conducted in the absence of any commercial or financial relationships that could be construed as a potential conflict of interest.
