# Supplementary material for: CCL7 Is a Negative Regulator of Cutaneous Inflammation Following Leishmania major Infection
Source: Front Immunol. 2019 Jan 8;9:3063. doi: 10.3389/fimmu.2018.03063 (PMC6331479; doi:10.3389/fimmu.2018.03063)
Supplement: Supplementary file 1 [file Data_Sheet_1.PDF]

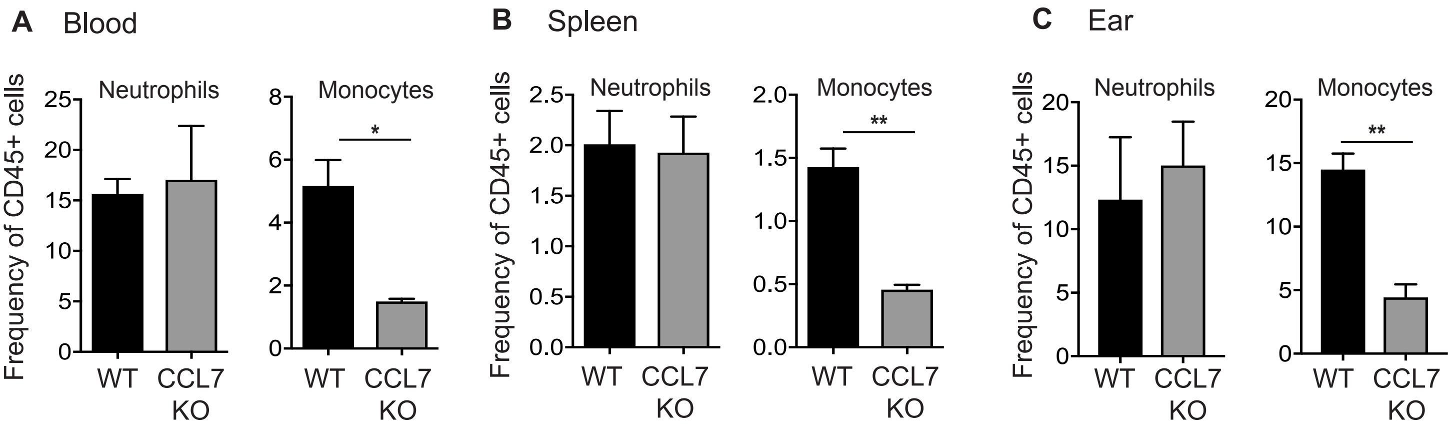

Supplementary Figure 1. Steady state frequencies of monocytes and neutrophils in CCL7-deficient mice. Analysis of monocyte and neutrophils frequencies in the blood (A), spleen (B) and ear skin (C) of naive (un-infected) WT and CCL7 KO mice. 4 mice per group, representative data from one of two independent experiments. WT, C57BL/6; KO, CCL7-/- C57BL/6 mice. Statistics by unpaired t test: *<0.05, **<0.01.

Supplementary Figure 2

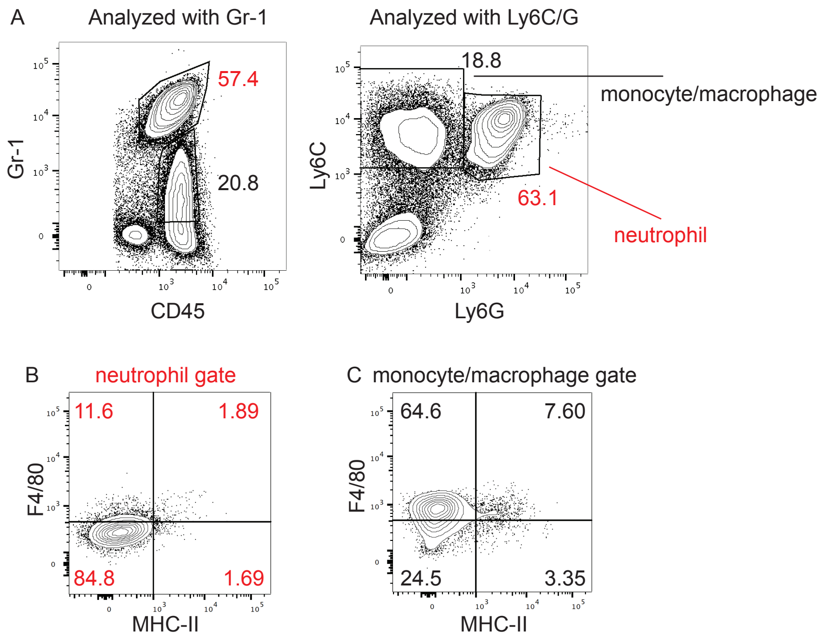

Supplementary Figure 2. Phenotyping of myeloid populations. (A) Myeloid cells from the *L. major* infected ears of CCL7 KO mice were phenotyped by FACS using Gr-1 or Ly6C and Ly6G. A sample from an individual mouse was stained with either Gr-1 or Ly6C/G for comparison. Left panel, representative FACS plot of Gr-1 staining. Right panel, representative FACS plot of Ly6C/G staining. Numbers are % of CD45+ cells, gated neutrophil population in red, monocyte/macrophage population in black. Both neutrophil and monocyte/macrophage populations were also positive for CD11b. (B) (C) F4/80 and MHC Class II expression on the cells in the neutrophil gate (B) and on cells in the monocyte/macrophage gate (C). Numbers in quadrants are % of gated population.

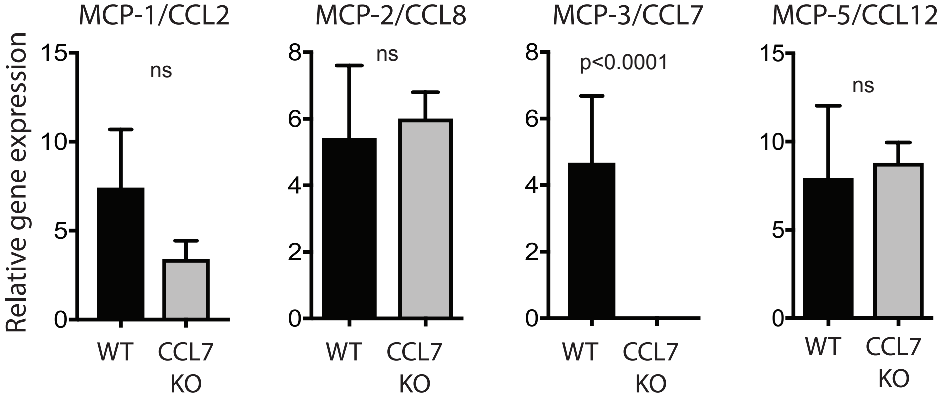

Supplementary Figure 3. No compensatory increase in MCP family members following *L. major* infection of CCL7 deficient mice. mRNA was isolated from whole ear extracts of naive and *L. major* infected (Day 14) WT and CCL7 KO mice and induction MCP genes tested using standard RT-PCR. 4 mice per group, representative data from one of two independent experiments. Statistics by 2-tailed T test.

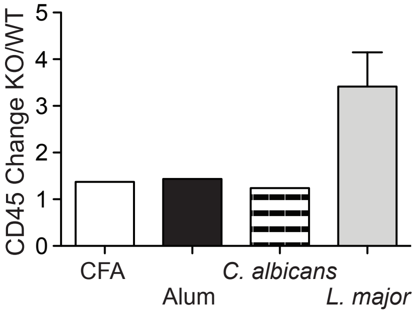

Supplementary Figure 4. ***L. major*-selective enhancement of inflammation in the absence of CCL7.** WT and CCL7 KO mice were infected in the dermis with *L. major* or a variety of other inflammatory stimuli. CD45+ cell inflitration into the ear was assessed at various timepoints post-immune challenge by flow cytometry. Analysis was perfromed at the peak of immune inflitration in the skin calculated for each individual stimuli. Mice immunized with CFA/OVA and Alum/OVA were analyzed 4-7 days after immunization when WT mice exhibited robust immune infiltration. Mice were infected with 10^6 *C. albicans* in the ear and analyzed 12 hr post-infection. The numbers of CD45+ cells in the ear were calculated by flow cytometry and data expressed as ratio of CD45 numbers between CCL7 KO and WT mice. Only inflammation in the context of *L. major* infection exhibited an increase in leukocyte numbers.

Supplementary Figure 5. **Gene list for custom Taqman Low Density Array (TLDA).** Genes analyzed for expression in WT and CCL7 KO skin following *L. major* infection.

Gene Symbol	Assay ID	Gene Symbol	Assay ID	Gene Symbol	Assay ID
18S	Hs99999901_s1	Csf2	Mm00438328_m1	Il3	Mm00439631_m1
Arg1	Mm01190441_g1	Ctla4	Mm00486849_m1	Il4	Mm00445259_m1
Bax	Mm00432050_m1	Cxcl10	Mm00445235_m1	Il5	Mm00439646_m1
Bcl2	Mm00477631_m1	Cxcl11	Mm00444662_m1	Il6	Mm00446190_m1
Bcl2l1	Mm00437783_m1	Cxcl9	Mm00434946_m1	Il7	Mm00434291_m1
Ccl1	Mm00441236_m1	Cxcr3	Mm00438259_m1	Il9	Mm00434305_m1
Ccl11	Mm00441238_m1	Ebi3	Mm00469294_m1	Itga4	Mm00439770_m1
Ccl19	Mm00839967_g1	Epx	Mm00514768_m1	Itgal	Mm00801807_m1
Ccl2	Mm00441242_m1	Fas	Mm00433237_m1	Itgb1	Mm01253227_m1
Ccl3	Mm00441258_m1	Fasl	Mm00438864_m1	Itgb2	Mm00434523_g1
Ccl5	Mm01302428_m1	Foxp3	Mm00475156_m1	Lta	Mm00440227_m1
Ccl7	Mm00443113_m1	Fut7	Mm01330673_g1	Ncf1	Mm00447921_m1
Ccr2	Mm99999051_gH	Gapdh	Mm99999915_g1	Nos2	Mm00440485_m1
Ccr3	Mm01216172_m1	Gata3	Mm00484683_m1	Plxna1	Mm00501110_m1
Ccr4	Mm00438271_m1	Gcnt1	Mm02010556_s1	Prf1	Mm00812512_m1
Ccr5	Mm01216171_m1	Gzmb	Mm00442834_m1	Retnla	Mm00445109_m1
Ccr7	Mm00432608_m1	Hprt1	Mm00446968_m1	Rorc	Mm01261022_m1
Cd19	Mm00515420_m1	Icos	Mm00497600_m1	Sele	Mm00441278_m1
Cd28	Mm00483137_m1	Ifng	Mm00801778_m1	Selp	Mm00441295_m1
Cd34	Mm00519283_m1	Il10	Mm00439616_m1	Sema3a	Mm00436469_m1
Cd38	Mm00483146_m1	Il12a	Mm00434165_m1	Sema4a	Mm00443140_m1
Cd3e	Mm00599683_m1	Il12b	Mm01288992_m1	Sema4d	Mm00443147_m1
Cd4	Mm00442754_m1	Il13	Mm00434204_m1	Sema6d	Mm00553142_m1
Cd40	Mm00441895_m1	Il15	Mm00434210_m1	Sema7a	Mm00441361_m1
Cd40lg	Mm00441911_m1	Il17a	Mm00439619_m1	Smad3	Mm00489637_m1
Cd68	Mm00839636_g1	Il18	Mm00434225_m1	Smad7	Mm00484741_m1
Cd80	Mm00711660_m1	Il1a	Mm00439620_m1	St3gal4	Mm00501503_m1
Cd86	Mm00444543_m1	Il1b	Mm00434228_m1	Tbx21	Mm00450960_m1
Cd8a	Mm01182107_g1	Il2	Mm00434256_m1	Tgfb1	Mm00441724_m1
Chi3l3	Mm00657889_mH	Il22;Iltifb	Mm00444241_m1	Timd2	Mm00506693_m1
Cma1	Mm00487638_m1	Il23a	Mm00518984_m1	Tnf	Mm00443258_m1
Csf1	Mm00432688_m1	Il2ra	Mm00434261_m1	Vcam1	Mm00449197_m1

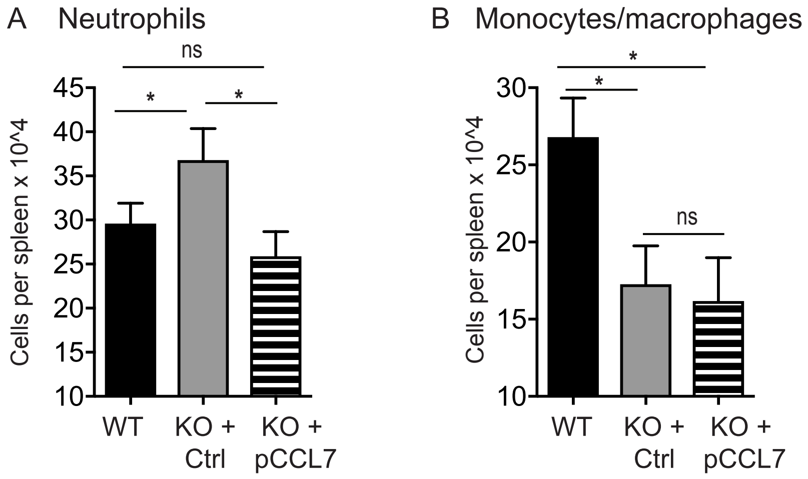

Supplementary Figure 6. CCL7 add-back reduces neutrophil accumulation in the spleen. Analysis of (A) neutrophil (PMN) and (B) monocyte/macrophage cell number in the spleen after *L. major* infection and CCL7 administration. Control, inactive (Ctrl), or active (pCCL7) CCL7 peptides were administered i.v. twice daily for 5 consecutive days starting day 9 post-infection and the spleen harvested 2 weeks post-infection. 3-4 mice per group, representative data from one of two independent experiments. WT, C57BL/6;KO, CCL7 deficient C57BL/6. Statistics by Mann Whitney.
